# Angular dependence of vortex instability in a layered superconductor: the case study of Fe(Se,Te) material

**DOI:** 10.1038/s41598-018-22417-3

**Published:** 2018-03-07

**Authors:** Gaia Grimaldi, Antonio Leo, Angela Nigro, Sandro Pace, Valeria Braccini, Emilio Bellingeri, Carlo Ferdeghini

**Affiliations:** 1 CNR SPIN, Salerno, Fisciano, 84084 Italy; 2 Physics Department, University of Salerno, Fisciano, 84084 Italy; 3 CNR SPIN, Genova, 16152 Italy

## Abstract

Anisotropy effects on flux pinning and flux flow are strongly effective in cuprate as well as iron-based superconductors due to their intrinsically layered crystallographic structure. However Fe(Se,Te) thin films grown on CaF_2_ substrate result less anisotropic with respect to all the other iron based superconductors. We present the first study on the angular dependence of the flux flow instability, which occurs in the flux flow regime as a current driven transition to the normal state at the instability point (I*, V*) in the current-voltage characteristics. The voltage jumps are systematically investigated as a function of the temperature, the external magnetic field, and the angle between the field and the Fe(Se,Te) film. The scaling procedure based on the anisotropic Ginzburg-Landau approach is successfully applied to the observed angular dependence of the critical voltage V*. Anyway, we find out that Fe(Se,Te) represents the case study of a layered material characterized by a weak anisotropy of its static superconducting properties, but with an increased anisotropy in its vortex dynamics due to the predominant perpendicular component of the external applied magnetic field. Indeed, I* shows less sensitivity to angle variations, thus being promising for high field applications.

## Introduction

The angular dependence of the critical current density *J*_*c*_, the upper critical magnetic field *H*_*c*2_ and the irreversibility field *H*_*irr*_ as a function of the orientation of the external applied field has been far and wide investigated in High Temperature Superconductors (HTS)^1^. A strong influence of anisotropy, layering and finite temperature on the transport properties of these attractive materials for potential applications is well recognized^2^. In HTS^1^ as well as in Iron Based Superconductors (IBS)^3^, the goal of increasing *J*_*c*_ and reducing their anisotropy passes through a deep understanding of a complex vortex matter and flux pinning landscapes. A critical issue for applications in high magnetic fields is the current carrying capability of these superconducting materials, which in the IBS case can even be surprising considering similarities and differences between the two classes of superconductors^4^. In particular, the elemental compound Fe(Se,Te) offers several advantages: few non-toxic elements, less complex layered crystal structure, and low anisotropy. Therefore, by assuming *θ* the angle between the parallel orientation in the *ab* planes and the direction of the applied magnetic field, a systematic study of the full current transport *J*(*θ*, *H*, *T*) in this material pave the way to set the ultimately limits to the performance of these IBS materials actual competitors of the HTS.

Historically, the HTS materials are classified as three-dimensional anisotropic or two-dimensional layered superconductors, on the basis that the coherence length in the crystal *c* direction, *ξ*_*c*_(*T*), exceeds the interlayer distance *s* or they nearly coincide, respectively^5^. It resulted that YBa_2_Cu_3_O_7±*δ*_ (YBCO) belongs to the former class, with an anisotropy parameter *γ* ≤ 10, whereas Bi_2_Sr_2_Ca_*n*−1_Cu_*n*_O_2*n*+4+*x*_ (BSCCO) to the latter one, with a *γ* > 10. As a consequence different types of vortex structures, such as pancake vortices or flux lines, and pinning mechanisms, as the intrinsic or the kink-pinning, have been established depending on the orientation of the applied external field^6^. Furthermore the vortex motion has been investigated by different external driving forces, either thermal or electric^7^, to get a comprehensive view of flux flow dissipations in these materials.

In the Fe(Se,Te) compound critical currents and its anisotropy have been explored since its discovery^8^, as well as in other IBS in connection with their multiband nature^9^. Typically the *J*_*c*_(*θ*, *H*, *T*) behavior shows a peak corresponding to the field orientations along the *ab*-planes, namely *ab*-parallel peak, and a peak corresponding to the *c*-axis direction, namely *c*-parallel peak; the former due to the intrinsic pinning^8^ or to the presence of planar defects^10^, whereas the latter is strictly related to an extrinsic pinning mechanism dominated by material defects parallel to the *c*-axis, if present^11^. Moreover, to our knowledge, a direct comparison between intrinsic *ab*-plane pinning and correlated pinning due to planar defects has not been deeply investigated in this Fe(Se,Te) superconductor^12^, as it has been done in HTS materials^13^. Indeed a different behavior can be observed for the same Fe(Se,Te) compound grown on different substrates^14^, by tuning the intrinsic/extrinsic pinning mechanisms. In the search for high-performance high-field superconductors, a good candidate can be the Fe(Se,Te) grown on CaF_2_ substrate^15^, which shows the lowest anisotropy^16^, a very robust *J*_*c*_(*H*) dependence^17^, and a sufficient stability against quench under relatively high bias currents^18,19^. Additionally, in this compound the study of vortex dynamics has been recently performed at subcritical current values and in self magnetic fields^20^. However, in the presence of an external applied magnetic field and at high vortex velocities, we have established the intrinsic nature of a quenching mechanism of the superconducting state, known as flux flow instability, occurring above *J*_*c*_ in the current driven transition to the normal state^18^. Moreover, several dynamical vortex flow instabilities have been also analyzed in superconductors exhibiting a negative differential resistance^21^. Finally, just recently, a sophisticated imaging of ultrafast vortices has been realized at nanometer scale, thus exploring flux flow instabilities by a local probe^22^, too.

Here we focus on the angular dependence of the flux flow instability, in particular of the critical parameters that are the quenching current I* and the critical voltage V*, which identifies the upper limits of the flux flow regime, that suddenly is driven into the normal resistive state^23^. Our purpose is to study the anisotropy of the flux flow instability in an IBS material that is the Fe(Se,Te) compound epitaxially grown on CaF_2_ substrate (see Methods). On top of that, we evaluate the anisotropy factor *γ*_*J*_ defined by the *J*_*c*_ anisotropy, since in multiband materials the anisotropy parameters are different when they are derived from *J*_*c*_, *H*_*c*2_ rather than from the penetration depth anisotropy^9^. The low anisotropy values, ranging from 1 to 2, makes this Fe(Se,Te) superconductor different from the other IBS as well as HTS materials, despite the similar layered structure. We find that: (*i*) the critical current *I*_*c*_(*θ*) shows the *ab*-parallel peak at *θ* = 0° and *θ* = 180° commonly related to the intrinsic layered structure of two-dimensional superconductors, being *ξ*_*c*_(*T*) ≤ *s* with *s* the interlayer distance^8,24^, or to correlated planar defects; (*ii*) the observed flux flow critical voltage V* follows, as a function of the angle *θ*, the usual scaling of the anisotropic Ginzburg-Landau model^25^; (*iii*) surprisingly, the instability current I*(*θ*), typically greater than the critical current, does not follow any available theoretical prediction. In other words, on one hand, there is a layered structure in this material that influences both V* and *I*_*c*_, but does not affect I*, which gives an higher stability for a better performance in all those applications in which the switching current to the normal state is relevant. On the other hand, experimental data analysis emphasizes a fundamental aspect of the Fe(Se,Te) material similar to some HTS such as BSCCO^26^, but it demonstrates a relevant difference on the quenching current of the Fe(Se,Te) compound, which provides more efficient, robust and field independent electric current transport on the orientation of the magnetic field.

## Results

**Figure 1:**
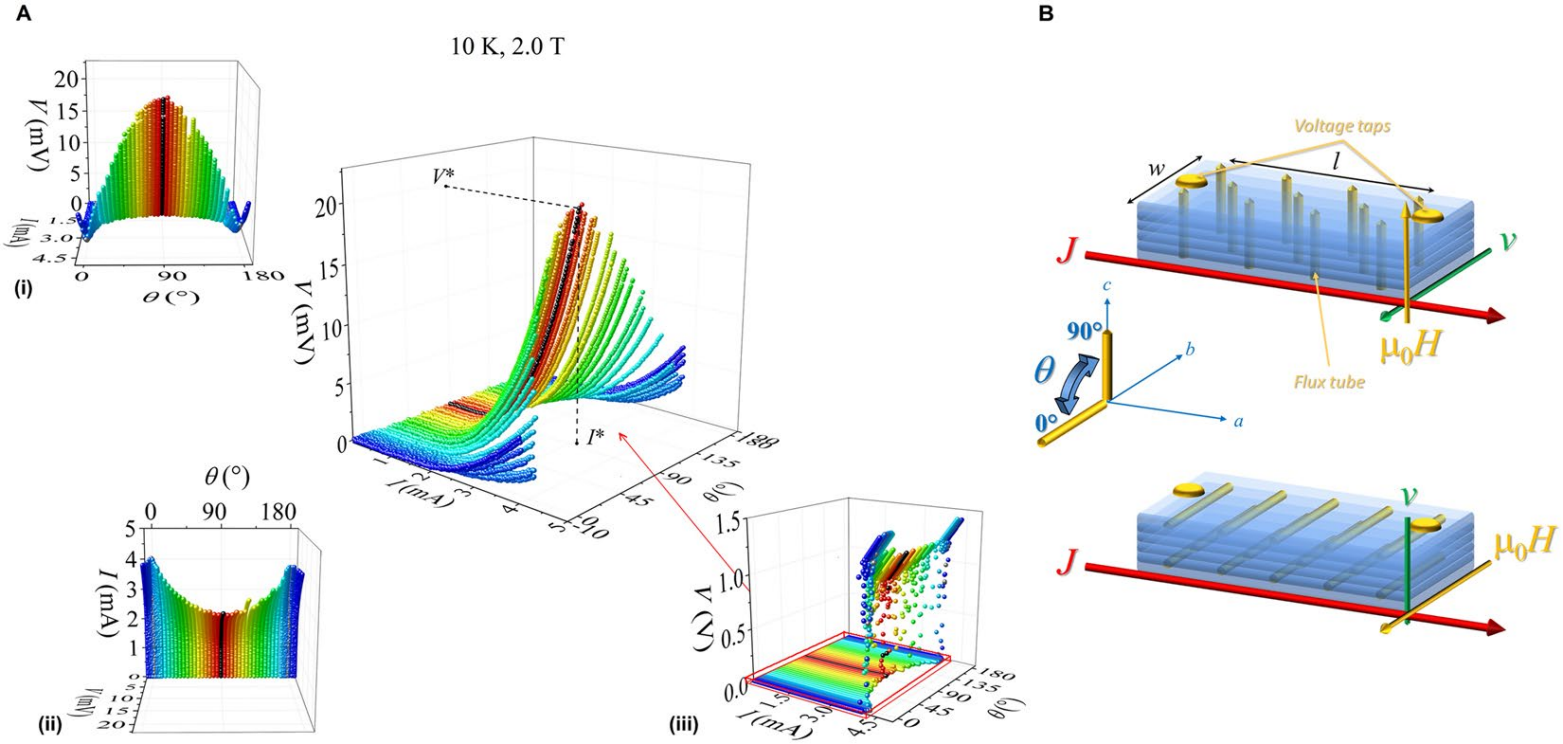
(**A**) Current-voltage characteristics at 2 T and 10 K as a function of the angle between the applied magnetic field and the direction parallel to the *ab*-planes. The individual angular dependences of *V* and *I* are shown (panels (*i*, *ii*)), as well as the (*I*, *V*) curves in the full scale up to the ohmic resistive branches (panel (*iii*)). (**B**) Schematic view of the sample geometry, with the indication of the orientation of the applied magnetic field *H*, the bias current density *J* and the resulting vortex velocity *v*. The yellow dots represent the voltage taps for voltage measurements as a function of the angle *θ*.

The phenomenon of flux flow instability and its angular dependence is studied as a function of the angle *θ* between the external magnetic field and the *ab*-plane at different temperatures and at several values of the applied field. The vertical external magnetic field direction is fixed and the sample is rotated so that the field goes from the direction parallel to *ab*-planes to the direction parallel to the *c*-axis, by always keeping the field perpendicular to the bias current, see Fig. 1B. Current-voltage *I*–*V* measurements are performed by a pulsed current bias technique, in an external field which is provided by an high field superconducting solenoid inside a cryogen-free magnet system (see Methods). Magnetic field-temperature *H*–*T* phase diagrams are acquired in the parallel ($\parallel$) and perpendicular ($\perp$) directions in order to extract the material physical parameters useful for the discussion (see Supplemental Materials for details). The microbridges of Fe(Se,Te) material epitaxially grown on CaF_2_ substrate (see Methods) have the following typical dimensions: thickness *d* of 150 nm, width *w* of 4 *μ*m, and length *l* of 50 *μ*m intended as the distance between the voltage taps, as displayed in Fig. 1B.

The flux flow instability consists in a quenching of the superconducting state, which abruptly drives the system from the dynamic non-linear flux flow resistive state into the normal resistance branch. Its fingerprint is essentially a voltage jump, marked by the critical parameters (I*, V*) in the current-voltage *I*–*V* characteristics, see Fig. 1A. This corresponds to a current driven transition, which may occur when a sufficiently high bias current destructively perturbs the flux motion out of equilibrium (see Supplemental Material for more details).

This phenomenon has been extensively studied in low temperature superconductors (LTS)^23,27–30^, as well as HTS^31–33^, and very recently in IBS^18,19^, too. Its angular dependence has previously been investigated in the BSCCO compound^26^, in which strong anisotropy is expected owing to its highly layered structure, i.e. *ξ*_*c*_(*T*) < *s*; while for YBCO the study focused on the in-plane angular dependence in connection with the symmetry of the order parameter^32^.

Our Fe(Se,Te) superconducting thin films grown on CaF_2_ substrates show different pinning contributions: one is anisotropic due to its layered structure or to possible planar defects although not revealed by TEM analysis^16^. Another pinning mechanism is ascribed to point-like randomly distributed isotropic defects, which is dominant at the lowest measured temperature of 4.2 K, that is induced by local modulation of Se and Te stoichiometry, with no *c*-axis correlated pinning^16^. Therefore, at increasing temperatures our Fe(Se,Te) films usually show an increasing anisotropy due to pinning. In fact, the critical current behavior becomes more and more anisotropic showing the *ab*-parallel peak more pronounced with respect to the flat angle dependence at lower temperatures^16^, as recently even confirmed by other authors^34^.

In addition, Fe(Se,Te) can also be considered an highly layered superconductor^8,24^, i.e. *ξ*_*c*_(*T*) ≤ *s*. Therefore, it is intriguing to observe how in dynamic conditions the flux flow instability depends on the angle variation, with a behavior that could be expected similar to BSCCO compound.

**Figure 2:**
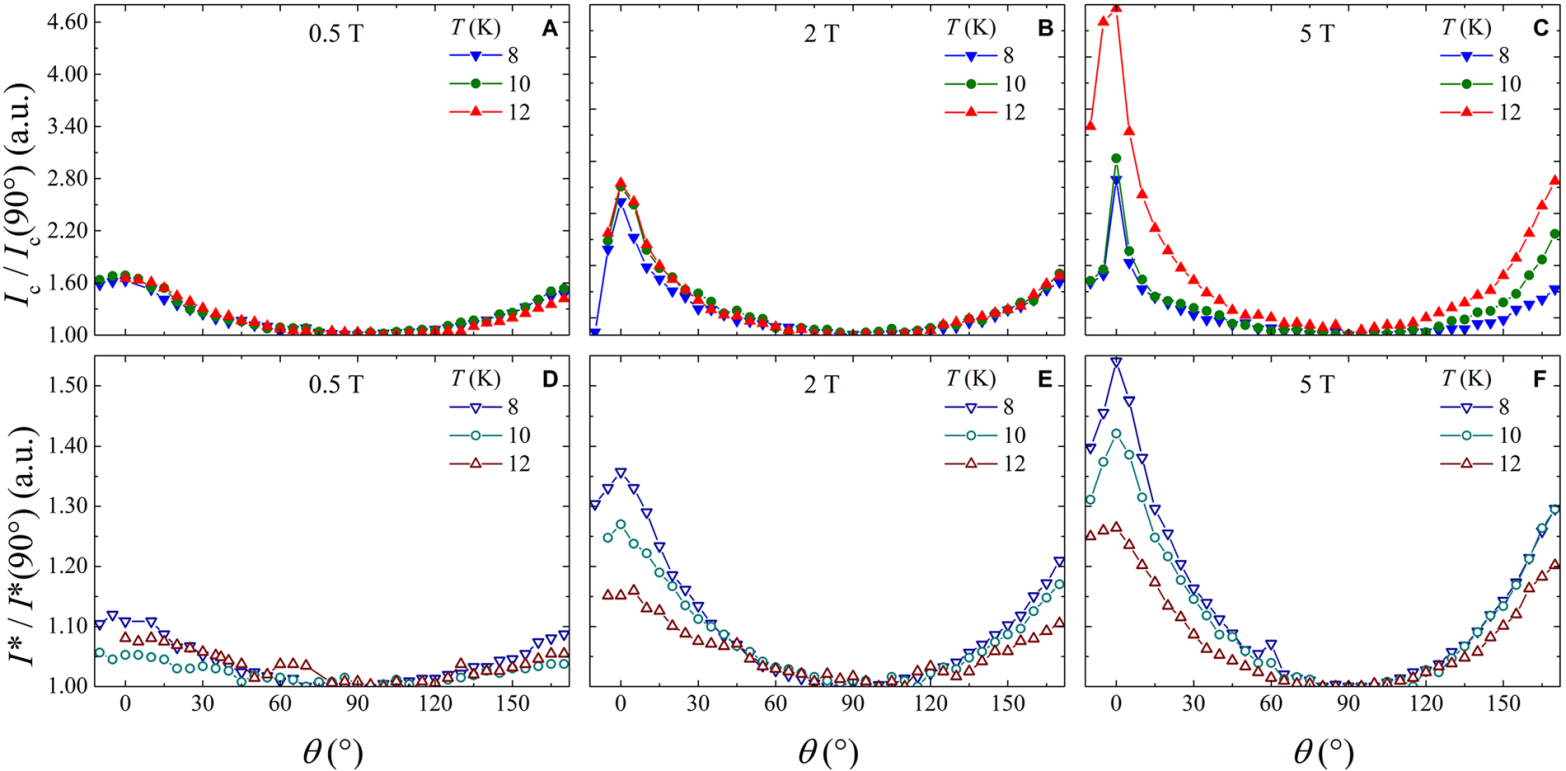
The critical current (panels (A–C)) and the instability current (panels (D–F)) as a function of the angle for different field and temperature values reported in the text. Both are normalized to the values corresponding to the field orientation perpendicular to the film surface.

**Figure 3:**
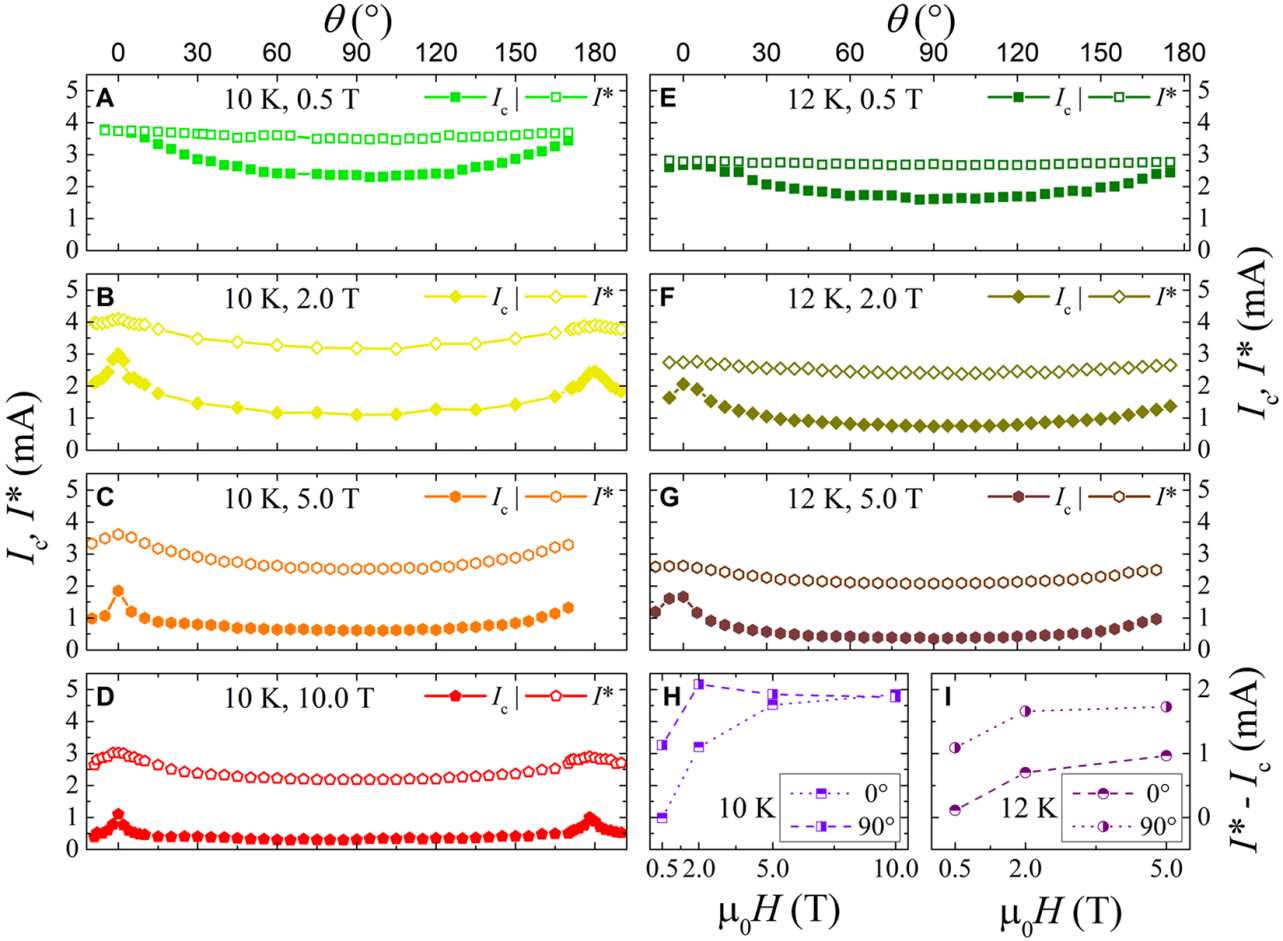
The comparison between the critical current and the instability current as a function of the angle for different fields at 10 K from panels (A–D), and at 12 K from panels (E–G). Panels (H,I) show the difference between the two current values as a function of the applied magnetic field for the two main orientation of the external field and the two measured temperatures of 10 K and 12 K.

Figure 1A shows typical *I*–*V* curves as a function of the angle *θ* at fixed temperature and at a field intensity value. The comparison of the angular dependence between the critical current and the instability current is reported in Fig. 2 for the three measured temperatures 8 K, 10 K, and 12 K, at different field intensities of 0.5 T, 2 T, and 5 T. The *I*_*c*_(*θ*) behavior reflects the angular dependence usually ascribed to the pinning influence of the layered structure of the Fe(Se,Te) material, as previously found, regardless of the substrate^8^. The *ab*-parallel peak is expected at *θ* = 0° and *θ* = 180° and they are actually observed. A scaling versus temperature is observed at all temperatures and low fields by normalizing the *I*_*c*_(*θ*) value to *I*_*c*_(90°), on the contrary this scaling is lost for the instability current I*(*θ*) at any temperature and field. Moreover, by increasing the magnetic field intensity, the *ab*-parallel peak in the *I*_*c*_(*θ*) becomes more and more pronounced, whereas the I* does not change so much. In any case no *c*-parallel peak is observed at *θ* = 90° that is a consequence of the homogeneous pinning landscape typically induced by the growth on this kind of substrate, as already established^16^. By a direct comparison of the *I*_*c*_(*θ*) with the I*(*θ*) values at fixed temperature and different fields in Fig. 3, it is possible to infer that the instability current is always greater than *I*_*c*_ and in the full measured magnetic field range, the critical current always drops by increasing field more rapidly than the instability current, whose dependence is quite robust. As a consequence, by increasing the applied magnetic field it results an increase of the difference between the instability and the critical current values, as displayed in Fig. 3H,I, regardless of the temperature. Anyway, a plateau is reached at very high fields, where a converging behavior is obtained no matter which is the field orientation.

**Figure 4:**
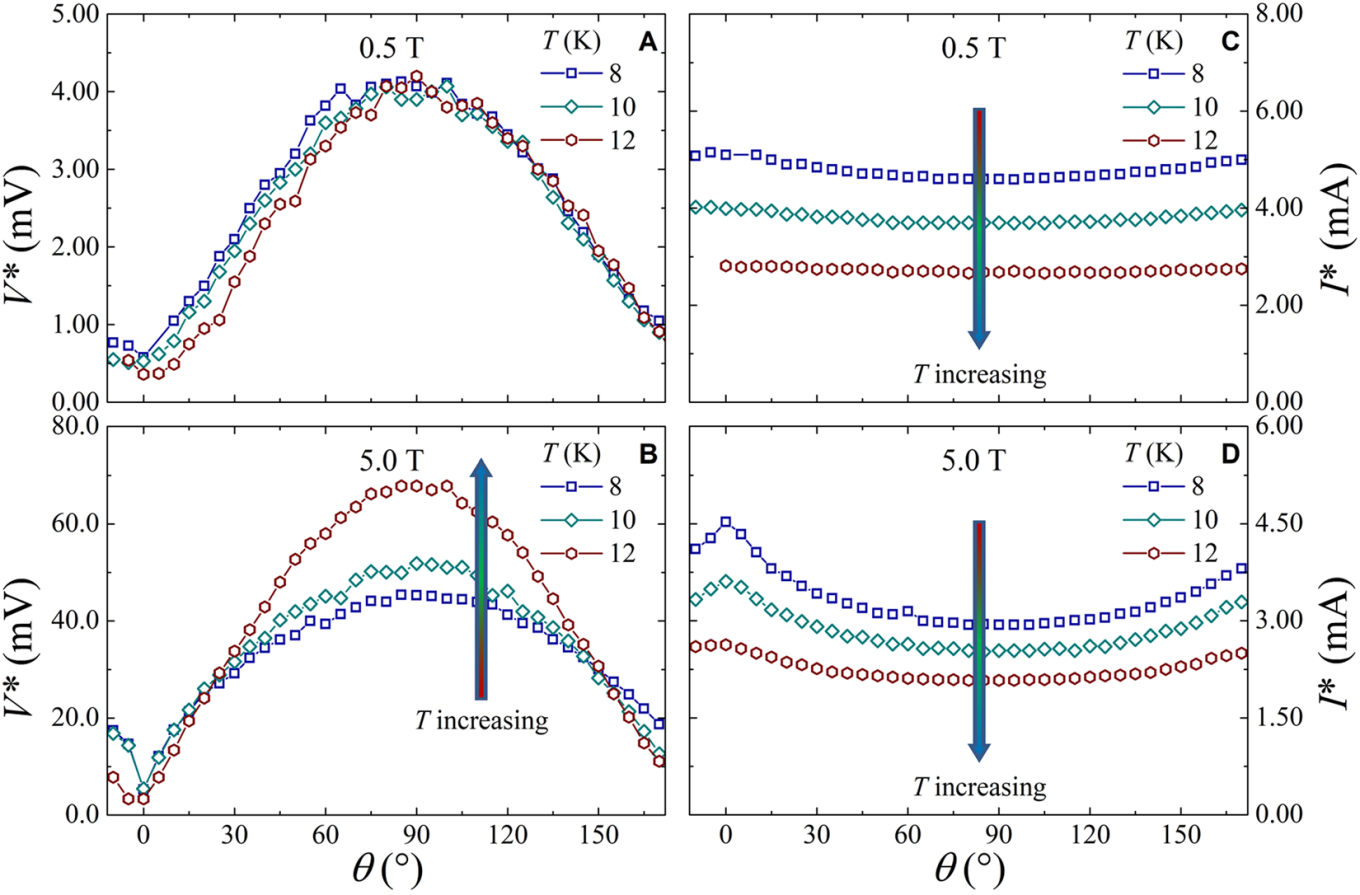
The critical parameters of the vortex instability, critical voltage V* and instability current I* as a function of the angle for the three measured temperatures of 8 K, 10 K, and 12 K and external field values of 0.5 T (panels (A,C)) and 5 T (panels (B,D)). The vertical arrows indicates the increasing direction of the temperature.

**Figure 5:**
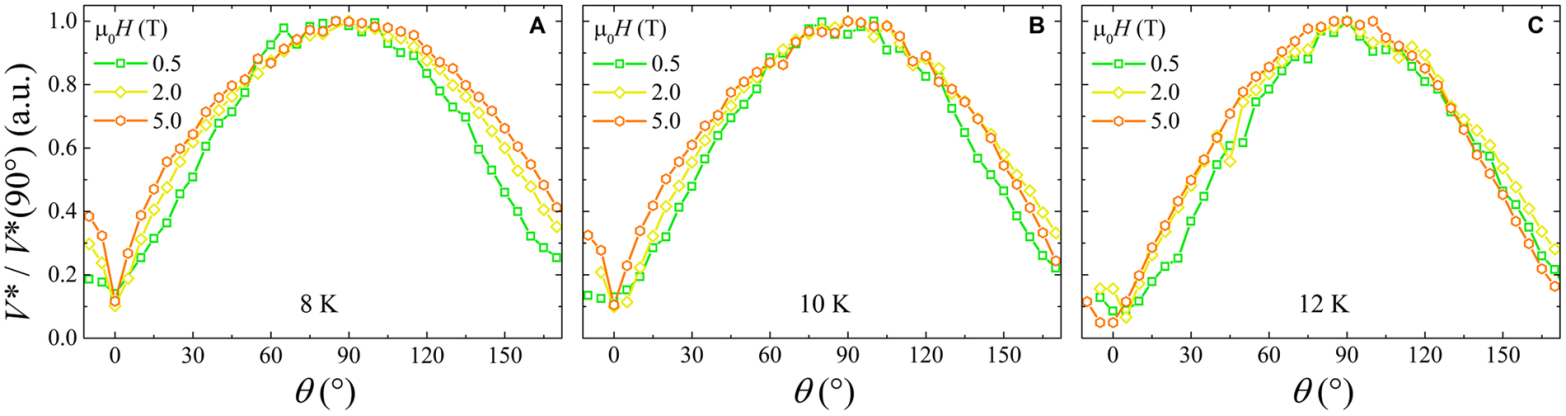
The critical voltage as a function of the angle normalized to the values corresponding to the perpendicular direction of the external field. The plots correspond to the full range of measured temperatures and magnetic fields.

The other critical parameter, which marks the instability of the flux flow state, is the critical voltage V*. The magnetic field and temperature dependence of this voltage is usually investigated in connection with the nature of the intrinsic or extrinsic mechanisms driving the transition to the normal state. In the Fe(Se,Te) superconductor, we have recently demonstrated that a coexistence of both mechanisms is possible^18^, and that this effect is highly influenced by the microbridge geometry^19^. In Fig. 4 the V*(*θ*) behavior is displayed at low and high field values, at the three different temperatures 8 K, 10 K, 12 K, in comparison with the corresponding I*(*θ*) dependence. It is worthwhile to remark on the opposite behavior of V* with respect to I*, since V* has a maximum at *θ* = 90° where I* shows a minimum, even if the former is well pronounced and the latter can be observed only at high fields. We definitely note that at low magnetic field the I* is much less sensitive to angle variation than V*. In addition, at high field V* increases as *T* increases, while I* decreases. However, by normalizing voltage as V*(*θ*)/V*(90°), an almost perfect scaling is found at different magnetic fields and temperatures, as displayed in Fig. 5. Thus, it is clear that the behavior of V*(*θ*) does not follow the same trend of I*(*θ*), and a different scaling rule has to be found in order to interpret this different angle dependences.

**Figure 6:**
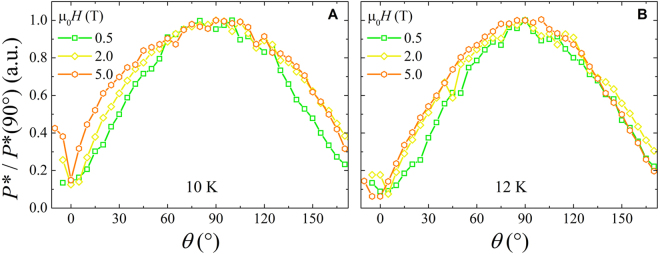
The dissipated electric power at the instability point as a function of the angle normalized to the values corresponding to the perpendicular direction of the external field. The plots correspond to the temperatures of 10 K (panel (A)) and 12 K (panel (B)) in the full magnetic field range investigated.

Furthermore the angular dependence of the electric dissipated power at the instability point P*(*θ*) = I*(*θ*) · V*(*θ*) is evaluated, with a scaling versus *T* and *H* similar to that of V*(*θ*), as shown in Fig. 6. This physical quantity is usually a measure of the heating effects, since a thermal runway rather than an intrinsic dominant trigger could affect the instability mechanism^18^. The angular variation of P* follows strictly the angular variation of V*, by analogy with the BSCCO case^26^, but in our case it is mainly due to the blindness of I* to the same angle variation.

## Discussion

The impact of flux flow instability on the transport properties of the superconductor has a counterpart on the current driven vortex dynamics at high velocities (see Supplemental Material). Although we are used to correlate flux pinning with critical current, and flux motion with dissipation, by gradually increasing the bias current above *I*_*c*_ the vortex motion can reach a speed limit which is unavoidably affected by pinning mechanism itself^35^. Either due to material defects^4,36^, or to geometrical barrier^37,38^, pinning becomes crucial in flux flow motion at high velocities too. Can the intrinsic layered structure of this Fe(Se,Te) superconductor make feel its influence?

The experimental findings on the several material parameters investigated offer the following scenario: (*i*) *I*_*c*_(*θ*, *H*, *T*) shows the only *ab*-parallel peak, which can be interpreted as the fingerprint of the intrinsic layered structure, thus confirming previous results on this Fe(Se,Te) material^8^. (*ii*) I*(*θ*, *H*, *T*) is much less sensitive to angle variation, such that it cannot be formulated within existing theoretical approaches (see Supplemental Material). (*iii*) V*(*θ*, *H*, *T*) displays a strong angle dependence with a perfect scaling vs field and temperature, whose interpretation can be found in the framework of anisotropy theories^25,26^. (*iv*) P*(*θ*, *H*, *T*) exhibits a trend similar to V*(*θ*, *H*, *T*) that points to the fact that self-heating effects are negligible with respect to electronic ones^18^. Additionally, the chosen microbridge geometry based on width less than 20 *μ*m, as well as the bias current pulsed technique based on 2.5 ms pulse width, can support the common origin of the voltage jumps in all magnetic field orientations, thus in agreement with our recent study on the same material performed only in perpendicular field configuration^18,19^, in which the intrinsic electronic nature of such instability has been demonstrated.

The general problem of anisotropic superconductors has been solved for strong type-II single-band superconductors by the scaling approach of Blatter *et al*.^25^. The scaling rule can be formulated in our case in the following way: *O*(*θ*, *H*, *T*) = *s*_*q*_*O*′(*ε*_*θ*_*H*, *γT*), being *O* the observable quantity for which *O*′ is the isotropic corresponding observable quantity, with *s*_*q*_ = *ε* or *s*_*q*_ = 1/*ε*_*θ*_ for the observable quantities volume, energy, temperature or magnetic field, respectively. Here *ε* = 1/*γ* is the anisotropy factor, and ${\varepsilon }_{\theta }^{2}={\varepsilon }^{2}\,{\cos }^{2}\,\theta +{\sin }^{2}\,\theta$ is the scaling factor for magnetic field such that it is *H*_*eff*_ = *ε*_*θ*_*H*.

The anisotropy factor *ε* can be evaluated by the mass anisotropy ratio *γ*_*m*_ = (*m*_*c*_/*m*_*ab*_)^1/2^ or by the *J*_*c*_ anisotropy parameter ${\gamma }_{J}={J}_{c}^{\parallel ab}/{J}_{c}^{\parallel c}$, whose magnetic field and temperature dependences can be different since *J*_*c*_ may be affected by factors other than the intrinsic anisotropy^9^. In the strong pinning regime for this multiband superconductor it has already been proven that the scaling procedure for the critical current works by using the temperature dependence of the anisotropy parameter *γ*_*J*_^8^. Our estimate gives at lower temperatures an anisotropy value ranging from 1 to 2 (see Supplemental Material), which confirms the lower anisotropic pinning character of this material grown on CaF_2_ substrate^16^, more isotropic also with respect to the same superconductor grown on MgO^8^, as well as other layered 1111-IBS^4^, or 122-IBS^39,40^, too. Therefore in our case, it is possible to apply Blatter scaling rule to verify the anisotropy estimation for *γ*_*J*_ parameter from the magnetic field dependence of the critical current (see Fig. 3 in the Supplemental Material), as usually performed^8,34^.

On the other hand, in dynamic conditions, to describe the angular dependence of the flux flow critical parameters, an analogous approach with this anisotropy scaling could be applied (see Fig. 5 in the Supplemental Material), since we observe an evident angular variation in the magnetic field dependence of V*.

**Figure 7:**
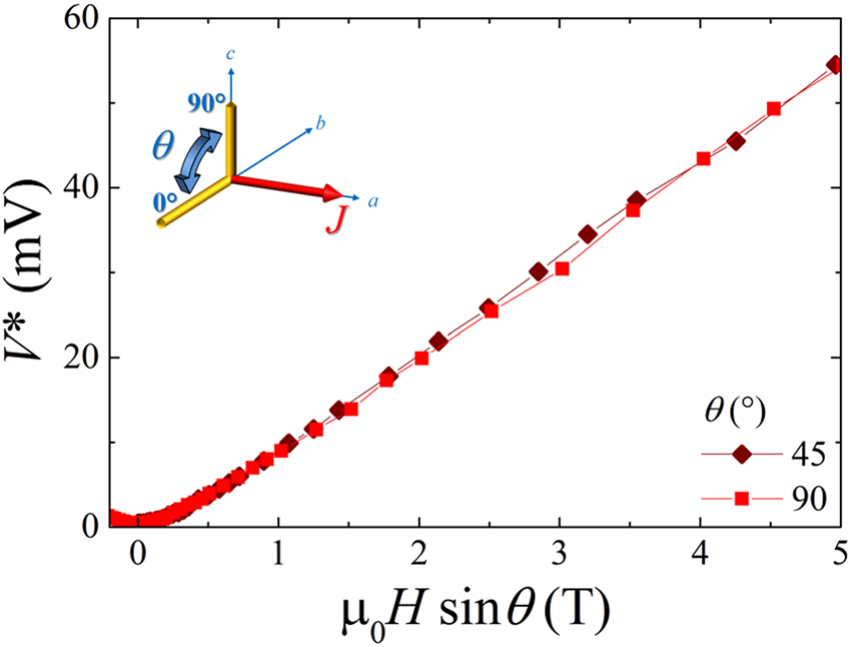
The scaling of the critical voltage as a function of the perpendicular component of the applied external magnetic field at *T* = 10 K.

Alternatively, it can turn useful the scaling approach followed by Xiao *et al*. in the case of the layered HTS BSCCO compound^26^. In fact, in Fig. 7 the magnetic field dependence of V* is plotted at different angles as a function of the field component perpendicular to the *ab*-plane *H* sin *θ* at *T* = 10 K. Data collapse on a single curve, that is the V* values are equal providing that *H* sin *θ* is considered. According to the observed scaling law, it is inferred that V* measurements are sensitive only to the *c*-axis component of the external applied field *H*, in analogy with the BSCCO case. Nevertheless, this is a further demonstration that Blatter scaling approach can be applied by supposing an higher anisotropy $\gamma \gg 1$ (see Fig. 5 in the Supplemental Material). In other words, the limiting behavior for large *γ* value of the highly anisotropic critical voltage observable V*(*θ*, *H*, *T*) = V*(*ε*_*θ*_*H*, *T*) = V*(*H* sin *θ*, *T*) reconciles both scaling approaches.

On the other hand, this effect can be expected on the general consideration of penetration of vortices in wide superconducting thin films, i.e. $w\gg d$. In tilted applied magnetic fields, it is the perpendicular component that penetrates in form of a vortex lattice, due to large demagnetization factor of thin films^26,41^. In a simplified scenario, two types of vortices can be distinguished: vortices that are perpendicular or parallel to the film plane. However, in a layered superconductor it is well known that a static staircase vortex configuration can be established depending on the tilting angle of the external applied magnetic field^4,13^. Moreover, our experimental findings for vortex dynamics show that the anisotropy of vortex instability originates from the layering nature, regardless of the geometry effects. Therefore, the dissipation is in practically only due to the component of the applied magnetic field perpendicular to the material layered structure, as in the case of the BSCCO compound extensively studied by Xiao *et al*.^26^.

On the basis of our experimental findings, we can argue that the Fe(Se,Te) is a challenging material that offers some unexpected potentiality, partially reflected in the different observed dependences of I*(*θ*, *H*, *T*) with respect to V*(*θ*, *H*, *T*). Indeed, the two superconductors, BSCCO and Fe(Se,Te), have in common at least the layered crystallographic structure so that they both could show a two-dimensional character, although the anisotropy factor is orders of magnitude different. In any case, this layering character influences the static and dynamics of Abrikosov vortices^4,35^. Consequently the layering nature of superconductivity is expected to affect the macroscopic quantity V*(*θ*, *H*, *T*) in a similar way in the two materials, as demonstrated in Fig. 7. Indeed, there is a direct correlation between the Abrikosov vortex velocity and the measured voltage V* (see also Supplemental Material), since by Maxwell equation V* = v* · *μ*_0_*H* · *l* (*l* is the distance between voltage pads).

Moreover, the different fabrication processes induce a variety of material defects acting as pinning centers in the two compounds that can also contribute to the different dependences of I*(*θ*, *H*, *T*) in the two materials. In fact, the macroscopic quantity of I* plays the role of the energy supplied to the vortices in the flux flow motion just before the instability takes place, so that any distribution of pinning centers as well as their interactions with the moving vortices can influence such an instability point. In fact, we have previously reported on a tunable pinning strength effect, for example, on I*(*H*) behavior^42^. In that case of *Al* superconducting film with underneath magnetic pinning, we observed a different behavior of I*(*H*) and V*(*H*). In particular, despite the fact that *I*_*c*_ was affected by pinning influence, I* resulted practically *insensitive to changes in pinning strength*^42^, but not V*(*H*) that followed the expected trend as a function of pinning strength. In the absence of a complete theoretical description of flux flow instability, able to take into account not only the material pinning influence but also the multiband peculiar nature of this material, we cannot exclude that the multiband character of Fe(Se,Te), rather different from BSCCO, may influence the electronic mechanism at the base of such instability. Therefore, the present paper may stimulate future works concerning the phenomenon of flux flow instability in IBS materials, since the 11 family already shows remarkable differences with respect to HTS materials, despite their layered similar structures.

By summarizing, the Fe(Se,Te) is characterized by a low anisotropy both in the critical currents and in the magnetic critical fields, although it is a layered superconductor which shows a dynamic behavior much more anisotropic and similar to the cuprates such as the BSCCO material. This result can be interpreted by means of the pinning mechanism acting in the Fe(Se,Te) material. Indeed, at low temperatures and low fields, the pinning centers act isotropically inducing a quite insensitive field dependence of *I*_*c*_. By increasing the temperature and field this anisotropy increases. Above *I*_*c*_ and below I*, from the static staircase configuration of vortices through the layered structure, we pass to a dynamic regime where the dissipation becomes mainly due to the perpendicular component of the applied field. Thus a further increased anisotropic behavior in vortex dynamics is induced. Finally, the instability current I* also reflects the *ab*-parallel peak symmetry of the layered structure, although it results less sensitive than the critical current.

In conclusion, the Fe(Se,Te) material has some similarities with respect to the HTS materials, but the I* dependence on the angle variation is very smooth in comparison with that found for example in BSCCO. This is indeed another case in which the instability voltage V*(*θ*, *H*, *T*) and the instability current I*(*θ*, *H*, *T*) are affected by the pinning mechanism^36,42,43^. However, I* turns to be more robust and isotropic in IBS rather than in HTS, thus promoting this material as an high-field superconductor.

## Methods

### Transport measurements

Measurements were performed in a Cryogenic, Ltd. Cryogen-Free Measurement System equipped with a variable temperature insert operating in the range 1.6 to 300 K with a temperature stability of 0.01 K, and equipped with a superconducting magnet able to generate up to 16 T. *I*–*V* characteristics were acquired by a pulsed current operation mode specifically developed to minimize self-heating effects. In particular, rectangular pulses of duration 2.5 ms with an interpulses current-off time of 1 s was used. The sample temperature has been monitored during each *I*–*V* acquisition, which were recorded by increasing and decreasing bias current: no thermal hysteresis has been observed. Data collected as a function of angle variation are obtained by a double-axis rotator probe, on which the sample was mounted with the bias direction always perpendicular to the orientation of the magnetic field. *H*–*T* phase diagrams were obtained by *R*(*T*) measurements performed with the sample thermally coupled to a copper block in flowing He gas.

### Sample fabrication

Thin films of Fe(Se,Te) epitaxially grown on CaF_2_ substrates were obtained by pulsed laser deposition from a FeSe_0.5_Te_0.5_ target with a Nd:YAG laser at 1024 nm. High quality and purity thin films were deposited at 550 °C, on which several microbridges were realized by standard UV photolithography and Ar ion-milling etching. The typical geometry consists of bridges few microns wide, which are all 50 *μ*m long. The nominal thickness of the films is 150 nm. Two identical series of microbridges were obtained on the same thin film.

## Electronic supplementary material

Supplementary Information
