# Supplementary material for: Angular dependence of vortex instability in a layered superconductor: the case study of Fe(Se,Te) material
Source: Sci Rep. 2018 Mar 7;8:4150. doi: 10.1038/s41598-018-22417-3 (PMC5841287; doi:10.1038/s41598-018-22417-3)
Supplement: Supplementary file 1 — Supplementary Information [file 41598_2018_22417_MOESM1_ESM.pdf]

[Supplementary Information] Angular dependence of vortex instability in a layered superconductor: the case study of Fe(Se,Te) material

Gaia Grimaldi[1,*,+], Antonio Leo[2,1,+], Angela Nigro[2,1], Sandro Pace[2,1], Valeria Braccini[3], Emilio Bellingeri[3], and Carlo Ferdeghini[3]

[1]CNR SPIN, Salerno, Fisciano, 84084, Italy
[2]University of Salerno, Physics Department, Fisciano, 84084, Italy
[3]CNR SPIN, Genova, 16152, Italy
[*]gaia.grimaldi@spin.cnr.it
[+]these authors contributed equally to this work

Flux Flow Instability

The instability of the superconducting state has been studied so far as an interesting phenomenon of vortex dynamics as well as for its relevant impact on the lossless electric current transport in type-II superconductors. No matter is the mechanism triggering the instability, its fingerprint consists of voltage jumps that can be observed in current-driven current-voltage characteristics of the superconducting material. Such jumps can be ascribed to several possible mechanisms, each of which shows its own peculiar feature in the $I-V$ curve branch above the critical current. Here we make a list of the conventional and more exotic ones in connection with their observable fingerprints. *Thermal runway*[4] is well known, since high currents induce a power dissipation in the film that is high enough to destroy the superconducting state, leading to an abrupt increase of sample temperature above T_c. *Hot spot effect*[4] is related to a localized normal domain (hot-spot) maintained by Joule heating, usually such domain appears where there is a maximum current concentration; the $I-V$ curve manifests an counterclockwise hysteresis. *Electron overheating*[1] is due to the finite heat removal rate of the power dissipated into the sample, depending on the film-substrate interface transparency to phonons, indeed non-equilibrium phonons leave the film without being reabsorbed; therefore the heat removal rate is determined by the strength of the electron-phonon coupling constant rather than by interface properties. *Vortex system crystallization*[5] may occur if the system has enough time to arrange itself into a coherently moving perfect crystal at large velocities; the ordering of the vortex lattice at large applied currents show a jumplike transition between pinned static state and homogeneously moving lattice. *Self-organized criticality*[9] is marked by voltage instabilities that could appear near the pinning-depinning transition by thermally activated jumps of vortices, which trigger a chain reaction of vortex movements leading to avalanches of diverging size. *Phase-slip centers*[11] (PSC) and/or *lines*[12] (PSL) occur at currents larger than a certain instability current I^*, a system of transverse alternating normal and superconducting domains is formed, and a voltage-step structure in the $I-V$ curve appears; these segments of constant dynamic resistance have a slope independent from the magnetic field strength. PSC appears when the uniform superconducting state is destroyed since the transport current reaches the GL pair-breaking current ($I_c^{GL} < I < I_{c2}$); PSL appears when the steady viscous flux flow of Abrikosov vortices is disrupted at currents $I_m < I_c^{GL}$. The normal state is reached at current higher than the upper critical current $I > I_{c2} \gg I_m$.

Theoretical approaches within this scenario include the fundamental *theory of Larkin and Ovchinnikov*[7] and the *hot electrons instability model* by Kunchur[6]. The first predicts the instability from the flux flow regime at temperatures close to T_c, caused by the shrinking of the vortex core due to the quasiparticles escaping when a sufficiently high vortex velocity is reached. The second main feature is the expanding of the vortex core as a consequence of the electronic temperature increase which adds quasiparticles within the vortex core at sufficient high electric field but at temperatures far from T_c. Nevertheless, both approaches ignores material pinning influence on flux flow instability, since originally they are derived in totally absence of any pinning mechanism. Moreover, just recently, pinning effects have been taken into account in the hot electron flux flow instability by Shklovskij[10].

We have recently demonstrated that some of the aforementioned instability mechanisms may compete depending on the material under investigation, so that in Fe(Se,Te) superconductor the flux flow instability can be considered halfway between

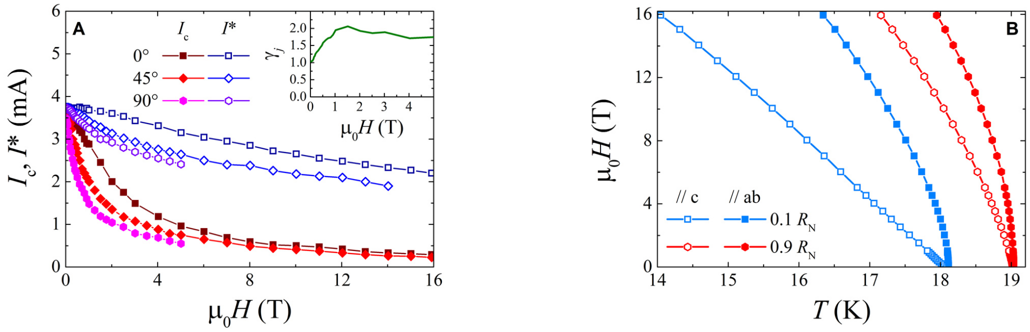

Figure 1. A. The critical current and the instability current as a function of the magnetic field intensity for three main orientation of the external field at $T = 10$ K. The inset shows the anisotropy parameter as deduced from the critical current measurements as a function of the field. B. The magnetic field-temperature phase diagram for the two main orientation of the external field.

those of LTS and HTS, with a coexistence of thermal effects dominated by the electronic nature of the instability[8].

Sample characterization

Pinning properties

Transport measurements were performed in order to characterize the pinning properties of this material and their anisotropy. Indeed, the anisotropy factor can be deduced from the critical currents vs field dependence measured in the two field orientation, that is the ratio of $I_c(0°,H)$ to $I_c(90°,H)$. In the Figure 1A the critical current versus H curves are displayed at different magnetic field orientations $\theta = 0°$, 45°, 90°, and at fixed temperature $T = 10$ K. The same plot includes the instability currents as well. It is clear that the I^* values are slightly dependent on the intensity and the orientation of the magnetic field. By the way, the I_c values reflects the expected behavior with the parallel in field values always greater than the perpendicular ones. Furthermore, the critical current density is $J_c = 2 \cdot 10^5$ A/cm^2 at 10 K. The inset shows the anisotropy factor γ_J versus field dependence, i.e. $I_c(0°)/I_c(90°)$, which results between 1 and 2 up to 5 T.

Anisotropy properties

The high quality of Fe(Se,Te) thin films is also confirmed by the $H - T$ phase diagrams measured in perpendicular and parallel orientation of the applied magnetic field, as reported in Figure 1B. In particular the critical temperature value T_c estimated by the 50% of the normal state resistance in zero field is 18.5 K, with a transition width below 1 K. The transition width is defined as the difference between the temperature values corresponding to 90% and 10% of the normal state resistance R_N. These are also the two criteria used to identify the upper critical field H_{c2} and the irreversibility lines, respectively, in the phase diagrams shown in Figure 1B. In Figure 2 the temperature dependence of the resistance is displayed at different angles from $\theta = 0°$ to $\theta = 90°$ with a field intensity of 2 T.

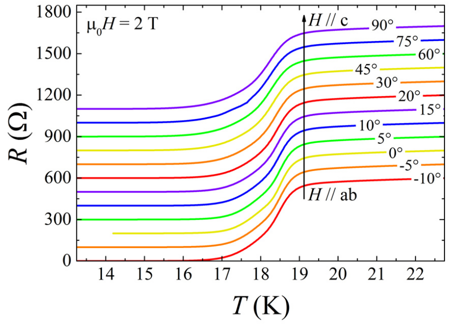

Figure 2. The temperature dependent resistance curves at 2 T for different angles from the field parallel to *ab*-planes up to the field parallel to *c*-axis. Curves are shifted by 100 Ω from each other.

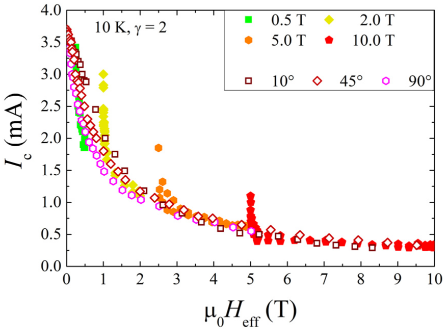

Figure 3. The Blatter's scaling curves of the critical currents with $\gamma = 2$ for different magnetic field intensities and for different field orientations at $T = 10$ K.

Vortex critical velocity

By rotating the external field from $\theta = 90°$ to $\theta = 0°$, the vortex velocity results from an in-plane vector velocity towards an out-of-plane vector velocity. In both cases, the resulting longitudinal electric field is detected from the two yellow dots used as voltage taps, as displayed in Figure 1B of the main text. The average vortex critical velocity is given by the measured critical voltage $V^* = v^* \cdot \mu_0 H \cdot l$. In Figure 4A, the $v^*(H)$ behavior is presented at different values of field orientation equals to 0°, 45°, 90°, and at the fixed temperature of 10 K. Clearly, in Figure 4B and C, the typical dependence of $v^* \propto H^{-1/2}$ is observed, which is the expected behavior of the intrinsic electronic nature of flux flow instability[3]. Moreover, it results that v^* increases from parallel (i.e. $\theta = 0°$) to perpendicular (i.e. $\theta = 90°$) orientation of the applied magnetic field. Consequently, in this latter case vortex lattice can move faster. In the inset of Figure 4A the corresponding pinning force dependence is also shown in order to enlighten that the stronger pinning results when the field is applied in the parallel direction, in agreement with the magnetic

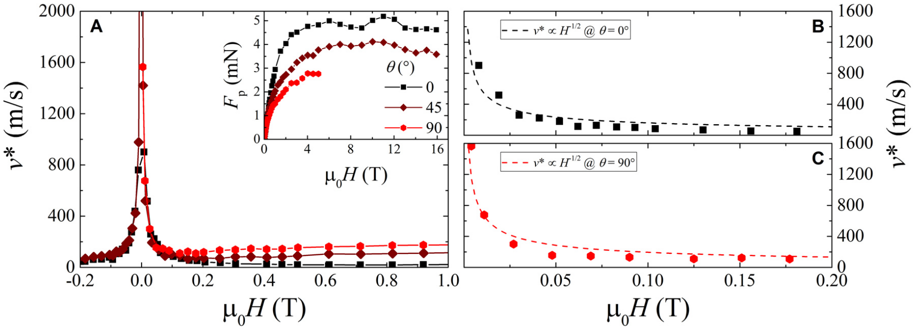

Figure 4. A. The vortex critical velocity as a function of the magnetic field intensity for three orientation of the external field at fixed temperature $T = 10$ K. The inset of panel A reports the corresponding pinning force values. B. shows the predicted typical trend (dashed lines) compared with experimental data at the two main orientation of the external magnetic field.

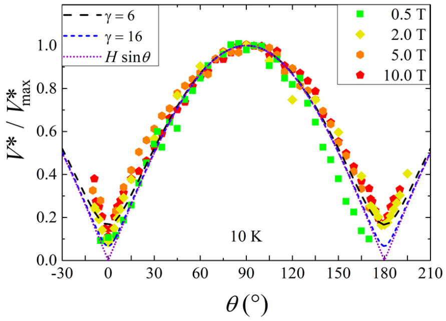

Figure 5. The critical voltage as a function of the orientation of the applied magnetic field at fixed temperature $T = 10$ K. The dashed lines show the predicted typical trends following Blatter's scaling approach with a different value of the anisotropy parameter γ, compared with experimental data at different intensities of the external magnetic field.

field dependence of the critical current (see Figure 1A). In addition we note that there is an increase of v^* vs H at increasing field intensity ($H > 0.1$ T) and for increasing angle orientation ($> 0°$). This is not the only case in which such unusual behavior can be observed[2]. Probably, this feature can be explained regardless of the specific material under investigation in a more general picture[10], but this deserves a more systematic study.

References

1. A. I. Bezuglyj and Shklovskij V. A. Effect of self-heating on flux flow instability in a superconductor near T_c. *Physica C.*, 202:234, 1992.

2. O. V. Dobrovolskiy et al. Pinning effects on flux flow instability in epitaxial nb thin films. *Supercond. Sci. Technol.*, 30:085002, 2017.

3. S. G. Doettinger et al. Electronic instability at high flux-flow velocities in high-T_c superconducting films. *Phys. Rev. Lett.*, 73:1691, 1994.

4. A. V. Gurevich and Mints R. G. Self-heating in normal metals and superconductors. *Rev. Mod. Phys.*, 59:941, 1987.

5. A. E. Koshelev and Vinokur V. M. Dynamic melting of the vortex lattice. *Phys. Rev. Lett.*, 73:3580, 1994.

6. M. N. Kunchur. Unstable flux flow due to heated electrons in superconducting films. *Phys. Rev. Lett.*, 89:137005, 2002.

7. A. I. Larkin and Y. N. Ovchinnikov. Nonlinear conductivity of superconductors in the mixed state. *J. Exp. Theor. Phys.*, 41:969, 1975.

8. A. Leo et al. Competition between intrinsic and extrinsic effects in the quenching of the superconducting state in Fe(Se,Te) thin films. *Phys. Rev. B*, 93:054503, 2016.

9. O. Pla and Nori F. Self-organized critical behavior in pinned flux lattices. *Phys. Rev. Lett.*, 67:919, 1991.

10. V. A. Shklovskij. Pinning effetcs on hot-electron vortex flow instability in superconducting films. *Physica C: Superconductivity and its applications*, 538:20, 2017.

11. A. G. Sivakov et al. Josephson behavior of phase-slip lines in wide superconducting strips. *Phys. Rev. Lett.*, 91:267001–1, 2003.

12. D. Y. Vodolazov and F. M. Peeters. Rearrangement of the vortex lattice due to instabilities of vortex flow. *Phys. Rev. B*, 76:014521, 2007.
